# A 390 million-year-old hyper-compound eye in Devonian phacopid trilobites

**DOI:** 10.1038/s41598-021-98740-z

**Published:** 2021-09-30

**Authors:** B. Schoenemann, E. N. K. Clarkson, C. Bartels, W. Südkamp, G. E. Rössner, U. Ryck

**Affiliations:** 1 Department of Zoology, Neurobiology/Animal Physiology and Biology Education, University of Cologne, Cologne, Germany; 2 Grant Institute, School of Geosciences, University of Edinburgh, West Mains Road, Edinburgh, EH9 3JW Scotland; 3 Kamen, Germany; 4 Altstrimmig, Germany; 5 Bayerische Staatssammlung für Paläontologie und Geologie, Richard Wagner Str. 10, 80333 Munich, Germany; 6 Department für Geo- Und Umweltwissenschaften, Ludwig-Maximilians-Universität München, Richard-Wagner-Str. 10, 80333 Munich, Germany; 7 Polling, Germany

**Keywords:** Evolution, Neuroscience, Physiology, Zoology

## Abstract

Trilobites, extinct arthropods that dominated the faunas of the Palaeozoic, since their appearance c 523 million years ago, were equipped with elaborate compound eyes. While most of them possessed apposition compound eyes (in trilobites called holochroal eyes), comparable to the compound eyes of many diurnal crustaceans and insects living today, trilobites of the suborder Phacopina developed atypical large eyes with wide lenses and wide interspaces in between (schizochroal eyes). Here, we show that these compound eyes are highly sophisticated systems—hyper-compound eyes hiding an individual compound eye below each of the big lenses. Thus, each of the phacopid compound eyes comprises several tens, in cases even hundreds of small compound eye systems composing a single visual surface. We discuss their development, phylogenetic position of this hyper-compound eye, and its neuronal infrastructure. A hyper-compound eye in this form is unique in the animal realm.

## Introduction

Trilobites are extinct arthropods that dominated the faunas of the Palaeozoic. The oldest functional structure of compound eyes described so far was that of the trilobite *Schmidtiellus reetae* (Bergström, 1973)^1^. It clearly resembles the apposition compound eyes of many diurnal insects and crustaceans living today. Besides this basic and most common type among trilobites, the so-called holochroal eye, there exists a second principle—the schizochroal eye in the suborder Phacopina. The facets are less numerous than in most holochroal eyes, but can reach diameters of 2 mm and more, and there are wide interspaces in between. Here, we show that below each of the these large lenses sits a small complete individual compound eye—so in total there results a hyper-compound eye, with several tens, in cases hundreds of compound eyes in one eye-system. This study is based on material of Wilhelm Stürmer, a pioneer of X-ray analyses in fossils during the seventies. Though unexplained until now, he found the relevant evidence, hitherto open-ended, and finally resolved in this study. In this form the hyper-compound eye of phacopid trilobites is probably unique in the animal realm, though similar if more complex, however, to the system of certain amphipod crustaceans. Furthermore, this ~ 400 million-year-old system suggests capacities of complex neuronal interconnections and processing, such as sharing of functions, perhaps colour perception, or neuronal superposition. These results may be an example of how studies of material, several hundreds of million years old, may reveal sophisticated visual systems, comparable in elegant complexity with those of today.

In this manuscript, we trace the historical sequence of knowledge acquisition during our study, which started to unravel the enigmatic filamentous structures in phacopid compound eyes that Wilhelm Stürmer had observed in the early seventies. Thus, firstly, we analyze and describe these filamentous elements—they are fossilized representatives of the efferent nerves of individual visual units. Secondly, basing on Stürmer’s X-ray documents and supported by computer tomography and synchrotron analyses we have been able to decipher the puzzling visual system of phacopid eyes referred to as hyper-compound eyes.

Wilhelm Stürmer (1917–1986) was head of the radiology department at Siemens corporation. Being a chemical physicist and radiologist, he combined these skills with his interests in palaeontology. To facilitate his palaeontological research, he bought a minibus, installed an X-ray machine within it, and between 1960 and 1986, travelled from quarry to quarry in the Hunsrück, part of the Central German uplands, and visited numerous collectors to investigate the faunas of dark-coloured slates, originally intended to be roofing tiles. Using soft X-rays (25–40 kV) and stereoscopic exposures combined with high-resolution films and image processing, he produced magnificent radiographs, not just of shells, but also of soft tissues, which in those times was a new dimension in palaeontology^2,3^.

The use of X-rays in palaeontology was begun by Broili^4,5^ and independently by Lehmann and others in the 1930s. In particular, Stürmer studied lightly pyritised specimens from the Lower Devonian Hunsrück Slate, of Bundenbach and Gemünd, Germany and somewhat similar material from the Ordovician Utica Slates of New York State. These studies revealed magnificent details of antennae, legs, gills, and intestines in the Devonian trilobites *Phacops, Asteropyge* (now *Chotecops*, resp. *Rhenops*)*,* and in the Ordovician *Triarthrus,* but in the German material some so far unknown, and unexpected structures were also discovered within the compound eyes of the phacopid trilobites. These structures, first noted by Lehmann in 1934^6^, and seen in several specimens, have the form of 'a more or less marked striated pattern extending from the eye inwards'^7^^, p. 113^, and it is this striated region, consisting of subparallel fibres that is the first subject of the present study. As with Stürmer and Bergström the filaments are here termed ' *fibers*'^7^^, p. 113^.

**Figure 1:**
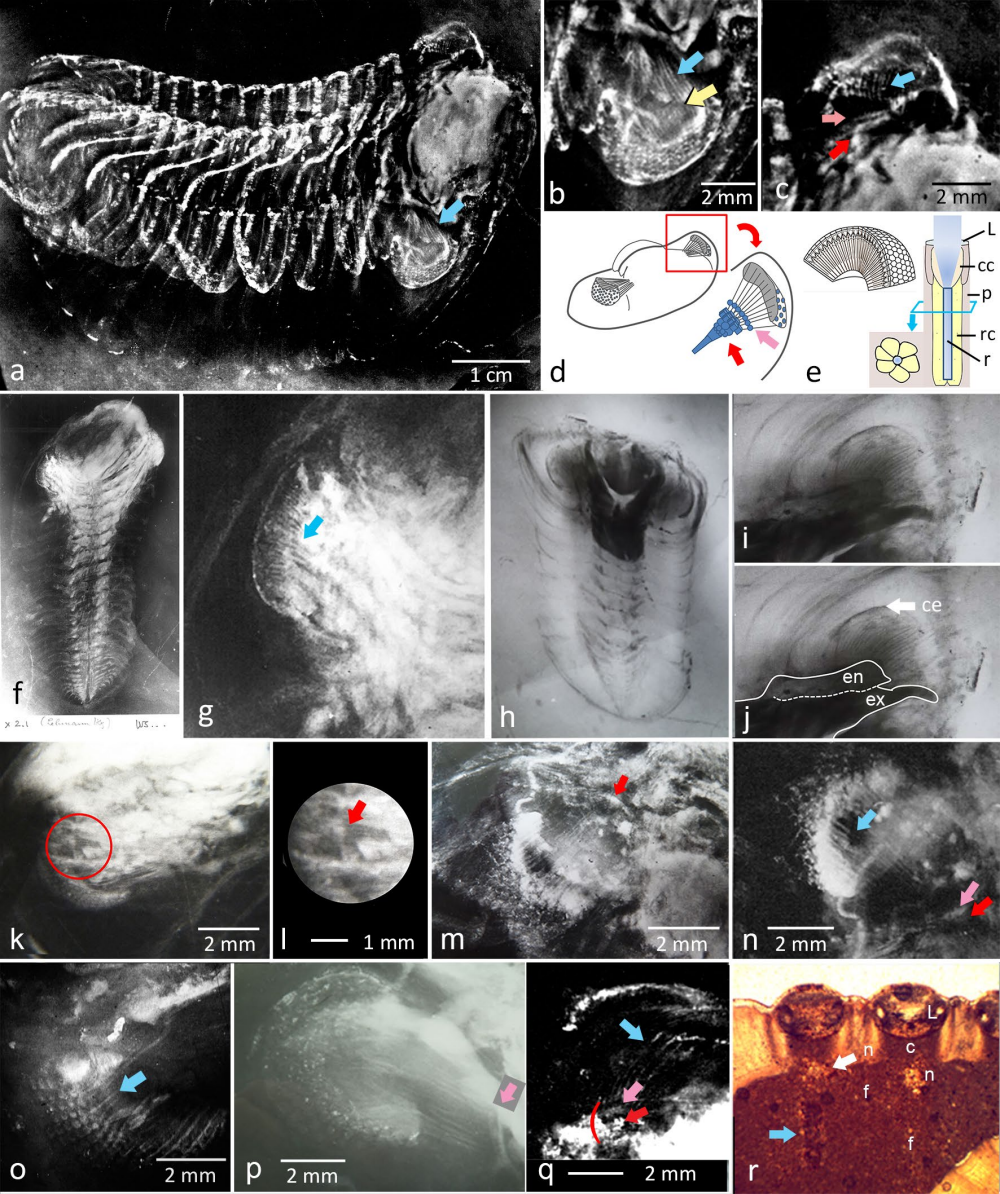
Filaments at the visual units of phacopid trilobites. (**a**) *Chotecops* (*Phacops*) *ferdinandi* (Kayser, 1880), [WS 2617]. (**b**) Right eye of a. ‘*fibers*' originating below the facets, converging towards interior of the cephalon. (**c**) Left eye of a, with (indistinct) ‘*fibers*', converging to plate of spheres and pyramidal element. (**d**) Schematic drawing of ‘*fibers*'. (**e**) typical ommatidium (apposition eye). (**f**) Lehmann’s specimen of *Asteropyge*^6^ (*Rhenops*) sp., [WS 2882]. (**g**) Left eye of f, showing ‘*fibers*'. h) *C. ferdinandi*, [WS 822]. (**i**) Appendage with gill-filaments, tapering distally. (**j**) i with endopodite (en) and exopodite (ex) of the appendage explained. (**k**) Eye of *C. ferdinandi*, each ‘*fiber*' connected to one visual unit [WS 4.1/503]. (**l**) Section of k, there as red circle. (**m**) Eye of *C. ferdinandi* [not numbered, XRCB], ‘*fibers*' converging to a centre. (**n**) *C. ferdinandi, ‘fibers*', converging to a supposed neuropile, [WS 295]. (**o**) *C. ferdinandi,* 1:1 relation between visual units; [WS 609(3)]. (**p**) *C. ferdinandi*, indistinct ‘*fibers*', converging to pyramidal element [WS 1832]. (**q**) *C. ferdinandi*, indistinct ‘*fibers*', converging to plate of spheres and pyramidal element [WS 1832]. (**r**) Thin-section of the eye of *Phacops tafilatelensis* Alberti 1983, showing lens, capsule that contains the receptors, foamy structure below (supposed neuropil), and ‘*fibers*', c capsule containing receptor cells, cc crystalline cone, ce compound eye, f ‘*fiber*', L lens, n neuropile, rc receptor cells, r rhabdom, (**g**–**k**) no scales given. XRCB Stürmer’s x-radiographs in the Steinmann Institute, Bonn. blue arrows: ‘*fibers*'; red arrows: pyramidal element; yellow arrow: underlying appendage; pink arrows: neural plate; white arrow: neuropil. Figure 1a–c are taken from Stürmer et al.^22^^, pl. iV, Fig 15a^ and Fig. 1 c,n from Stürmer and Bergström^7^^, plate 21b,c^.

To Stürmer and Bergström^7^^, p. 113^, it was very tempting to suggest that the striae represent internal eye structures, such as structures of the ommatidia. If they were, the ommatidia, for trilobites, were indeed surprisingly long, at least some 25 times their own diameter. The position of the inner termination of the linear structures would indicate that the optical ganglia and probably the whole anterior end of the brain lie fairly far backwards in the labrum^7,8^ (Fig. 1a,b). Stürmer and Bergström suggested that in *Chotecops* (*Phacops*) *ferdinandi* (Kayser, 1880) each of the fibres is an integral part of the schizochroal eye’s optical system, that it is connected to an individual lens, and that in this respect it could be a fibre-optic-system. They cited their predecessor: 'To Lehmann it seemed incredible what he saw as ' “the nerve bundle at an angle of 45° backwards to a place where the ganglion of the lower gullet is located.”'^7^^, p. 113^, but they believed, as was the general consensus of that time, that soft tissues such as nerves could not be preserved and traced in the fossil record. Equally, considering their X-ray photographs, they '… were sure that the fibres belong to a fibre optical system' [comparable to rhabdoms] 'as in the eyes of recent arthropods. A fibre optical system consists of an inner fibre with a higher refractive index than the shell'^7^^, p. 113^. It was considered as a light guiding system, whatever purpose it may have had. It was not until 8 years later, that Mike Land^9^ analyzed similar phenomena in the deep-sea amphipod *Phronima*, where the elongated structures are crystalline cones, which were first discovered and described by Exner in 1891^10^^, p. 30^.

Only a single specimen of a different trilobite genus, *Asteropyge* (now: *Rhenops*, Acastoidea, Phacopina), bearing similar internal structures in the eye was known (Fig. 1f,g), as described by Lehmann^6^. Strong doubts were expressed about the validity of Stürmer’s and Bergström’s interpretation by Clarkson, Campbell^11–14^, and working on the collection of the late Prof. W. Blind, by Bruton and Haas^15^. All of whom pointed to the dissimilarity between the nature of the fibres and that of any optical system known at that time. Clarkson^11^^, p. 441–442;^^12^^, p. 16^; ^13^^, p. 125^ and Campbell^14^^, p. 175^ believed that the striated region did not represent part of the optical system, but was a portion of the gill branch trapped under the eye and preserved within a protected closed micro-environment below. Most authors other than Stürmer considered that a relatively short ocellar capsule had lain below each lens of the schizochroal eye, floored by a flat layer of cells, in other words a retina^12,14,16,17^ There was considerable support for the retinal-capsule model by physicomathematical analysis of the lens optics^18–20^. Bruton and Haas believed that the striations were secondary rock cleavage^15^^, p. 60^. They discussed the presence in *Geesops sp.* of a mesodermal capsule below an eye lens and suggested this may have housed a retina with many photoreceptors, or a series of fused or extended rhabdoms^21^^, p. 352^.

It may be interesting to know, how this controversy ended. It is documented by Clarkson in the meanwhile famous 'Treatise'^13^^, p. 125^. Here it is mentioned that Bergström in a personal communication in March 1995 'pointed out that these supposed optical fibres form part of a series with filaments of the outer limb branches'^13^^, p125^, in other word gills. Accordingly, the original figures 5a, p. 115 and plate 17b^7^ became modified in the 'Treatise', and the ommatidia were omitted. The label 'ommatidia' finally became removed from the original version^13^^, fig. 88, p.101, fig. 94, p.107^. Wilhelm Stürmer, however, still believed that the fibers were integral part of the visual system of phacopid trilobites until he died in 1986 [W. Stürmer, pers. comm. with E.C. 1985].

The various strands of information available at present seem to be mutually contradictory, and it seems pertinent to us to try to resolve this conundrum. Accordingly, we have studied all the pertinent literature, and all of Stürmer’s unpublished photographs that were still available, these are preserved in the heritage of Stürmer’s X-ray documentations. Stürmer’s private archive was given to the care of his friend Dr. Christoph Bartels, who kindly made this material available for us. (Now the material is deposited at the Steinmann Institute, University of Bonn.).

## Analysis of hitherto unseen images

One of the most important specimens of this historical discussion, specimen WS 2617^22^ (Fig. 1a^22^, displays a right eye in which the elements are clearly shown (Fig. 1b). Here and in other specimens, they extend from the lentiferous surface of the eye, converging to disappear, below the axial furrow (Fig. 1a,b,k–q). As can be seen in other specimens, they end up in a layer of globular or cubical structures forming a kind of continuous plate (pink arrows in Fig. 1c,d,n,p,q), followed by a second that covers a conical element (red arrows in Fig. 1c,d,m,n,q. Below the filaments, lies a shadowy structure showing some articulation with a distinctive angle; suggestive of a bent appendage (Fig. 1a, yellow arrow in Fig. 1b), which probably gave rise to the idea that the elements were actually gill-filaments. The left eye of specimen WS 2617 also shows indications of these filamentary structures (Fig. 1c).

Another specimen of *Phacops* (WS 2822) (Fig. 1h–j) displays the gill appendage properly. It shows two components: at its end, firstly, a finger-like, slightly curving rod, showing up as black in the photograph, secondly at about two thirds of the width of the end of the appendage a flat structure that fringes out as an array of filaments. The filaments are broader proximally, tapering distally. These can only be interpreted as gill filaments at an exopodite of the leg, the finger-like part being the endopodite (Fig. 1i,j). On the left hand side of these gill filaments, the external contours of a compound eye can be clearly seen (white arrow Fig. 1j). These gill filaments taper distally, becoming finer and finer. It is the other way round with the ‘*fibers*'. They taper proximally, thus the elements under discussion cannot be gill filaments (Fig. 1k–o).

Stürmer and Bergström^7^^, plate 21^ documented several examples of compound eyes 'showing lenses and the ‘*fibers*' leading to the ganglion'. The most informative ones are repeated here (Fig. 1n,q), complemented by some others from the archive noted previously (Fig. 1a–c;^22^ m–q), and specimen WS 4.1/503 (Fig. 1k,l), (Hunsrück-Museum, Simmern). Especially the latter, but also others (Fig. 1k–o,q), nicely demonstrate that there is a 1 : 1 correlation between the visual units of the lenses and the ‘*fibers*' originating at the bases of these units (Fig. 1k–o,r). Clearly, the ‘*fibers*' are unbranched and straight. In Fig. 1k–o,r especially, it becomes evident that the ‘*fibers*' are wider at their origin, and taper proximally; this is quite different from situation in the gill filaments (Fig. 1i,j). Thus, our first result is that the ‘*fibers*' really exist, because are present in several specimens and species. They are not gill filaments, but are integral components of the compound eye, each being connected to one visual unit.

**Figure 2:**
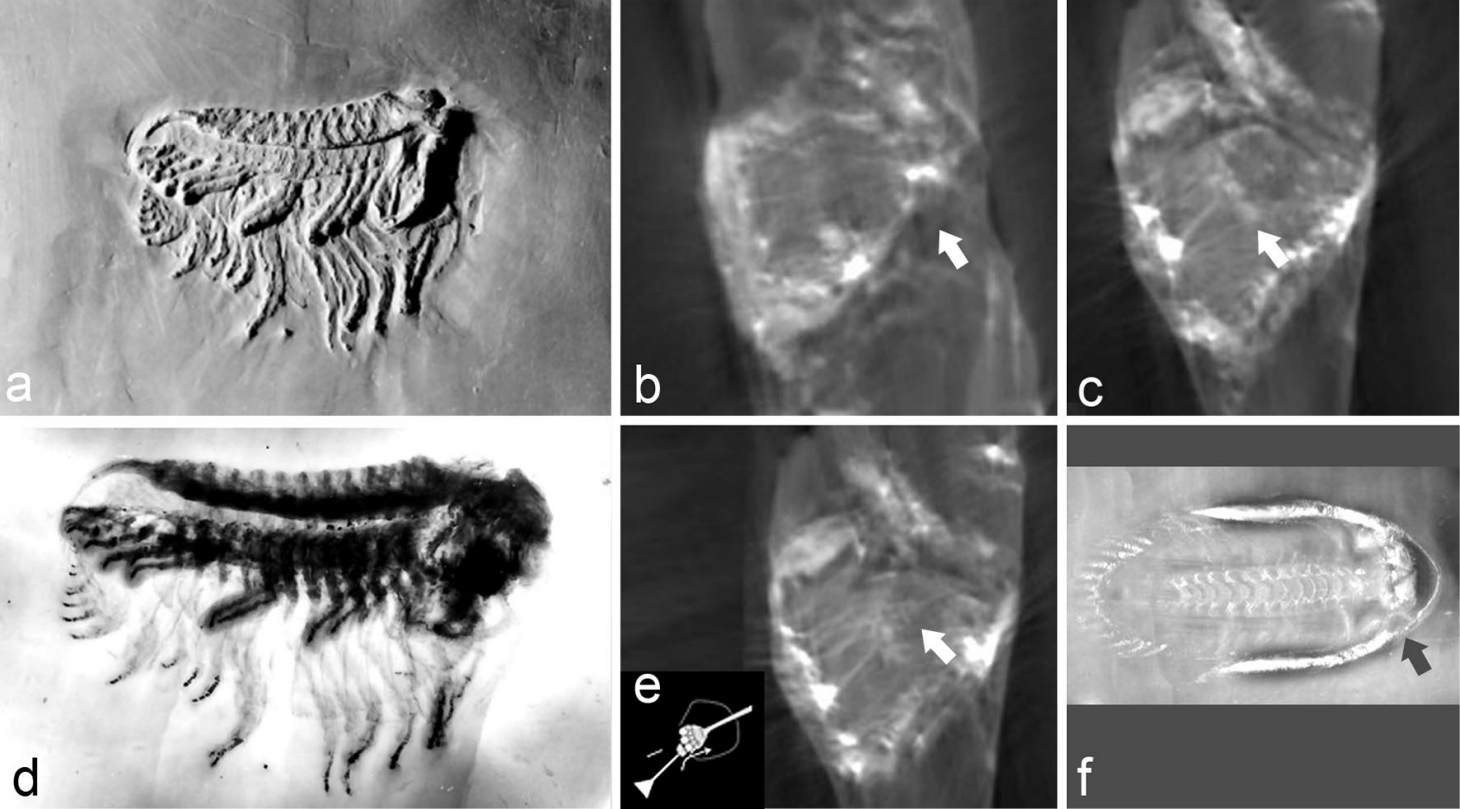
Interior structure of the eye capsule. (**a**) *Chotecops* (*Phacops*) *ferdinandi* (Kayser, 1880).WS 295 (SNSB-BSPG 1930 III 8). (**b**) ct of the right eye of a), note the tube at the distal end of the capsule (arrow). (**c**) Deeper section of (**b**), note the 'cube'-like structures below the '*fibers*'. (**d**) X-ray of (**a**), made by Stürmer. (**e**) Deeper section of (**c**), note the hierarchically ordered spherical structures (arrow) below of the 'cubes' as shown in (**c**), and the filamentous structure distally. (**f**) *Asteropyge* (*Rhenops*) sp. (WS 2236), showing an eye capsule (arrow), [this specimen was not provided with any scale].

Figures 1a,b,m,n,q excellently prove Stürmer’s idea that the fibres converge to a centre, though he had not actually found where this is located, or had not documented it. This centre shows a pyramidal shape (red arrow Fig. 1m), with a flat surface, oriented proximally. It seems to consist of a small, densely ordered array of small rectangular or spheroidal cubes to which the fibres insert (Figs. 1q, 2e). Slightly distally, the ' *fibers*'already have passed through a plate, consisting of globular structures also (pink arrows in Fig. 1c,d,p,q).

### The visual unit and its contents

**Figure 3:**
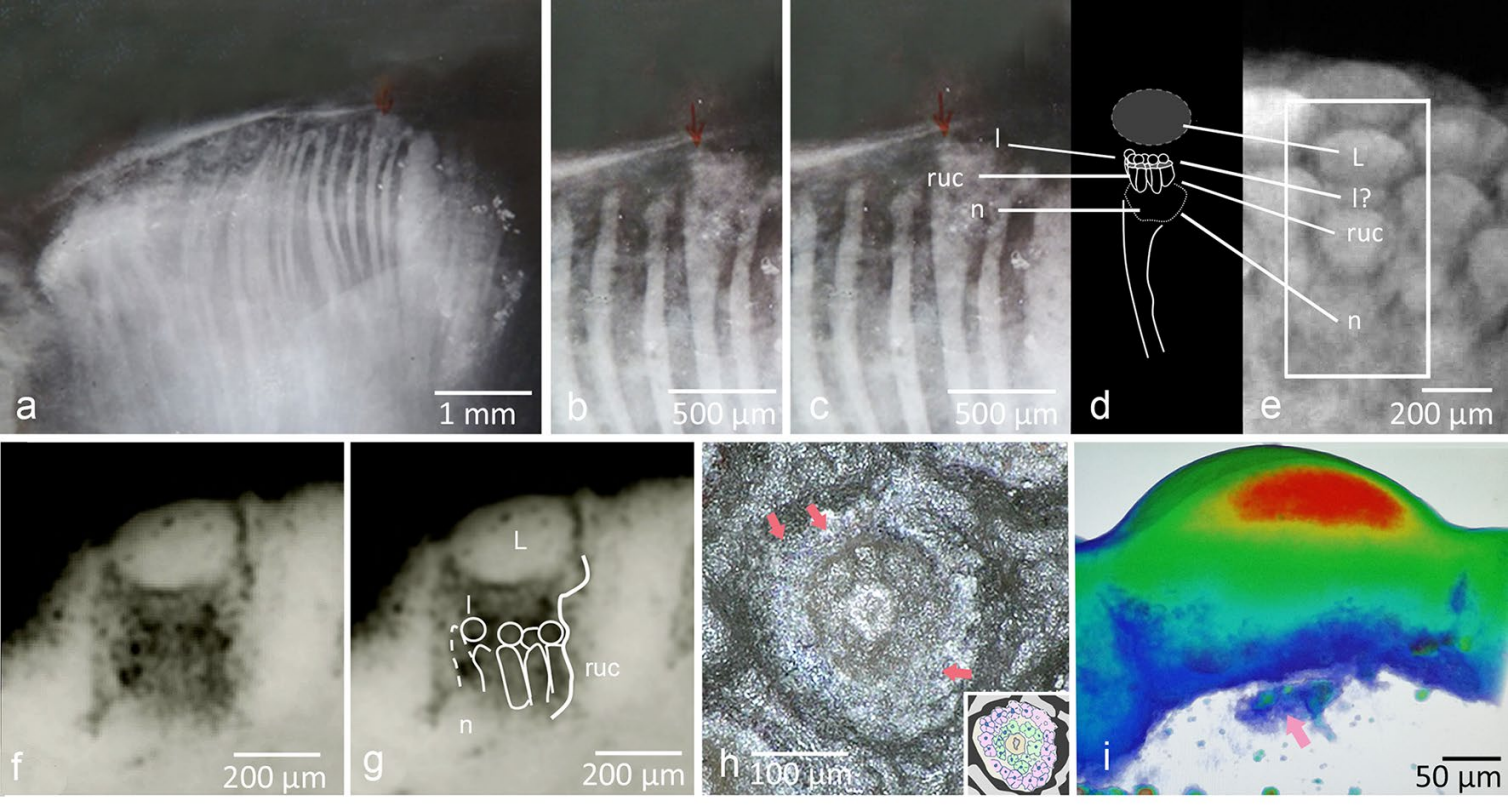
Structure of the hyper-compound eye of phacopid trilobites. (**a**) *Chotecops* (*Phacops*) *ferdinandi* (Kayser, 1880), [WS 11352c], X-ray of abraded compound eye with ‘*fibers*' and Stürmer’s red arrow. (**b**) Section of a. (**c**) Section of a, slightly brightened at the red-arrow-zone. (**d**) Schematic drawing of b. (**e**) µ-ct , *Geesops schlotheimi* (Bronn, 1825), Phacopida, showing a complete visual unit, comparable to those of *C. ferdinandi* in c. (**f**) Synchrotron radiation, longitudinal aspect of visual unit of *Barrandeops cf. granulops* Chatterton et al. 2006. (**g**) Interpretative drawing of f; small ommatidia hiding below a common wide lens. (**h**) *Phacops imitator* Struve, 1970; two concentric rows of ommatidia, pink arrows: rhabdoms. Insert: interpretative line drawing. pink: outer row of ommatidia, green: inner row of ommatidia, brown: central element, black: probably pigment, grey: cuticle (**i**) *G. schlotheimi*, synchrotron radiation, equal density colour coded, arrow: crown of sub-ommatidial lenses. L covering lens, l lenses of ommatidal sub-systems, ruc receptor unit’s complex, n neuropil.

In one of the folders containing a rich heritage of Stürmer’s X-ray photographs, we found a small negative showing an indistinct mark, an arrow drawn with red ink, though now almost invisible. When this is magnified, and the contrast enhanced, it goes a long way towards resolving Stürmer’s enigma (Fig. 3a–d). Even in a scientific paper, one has to congratulate Stürmer for this important discovery.

**Figure 4:**
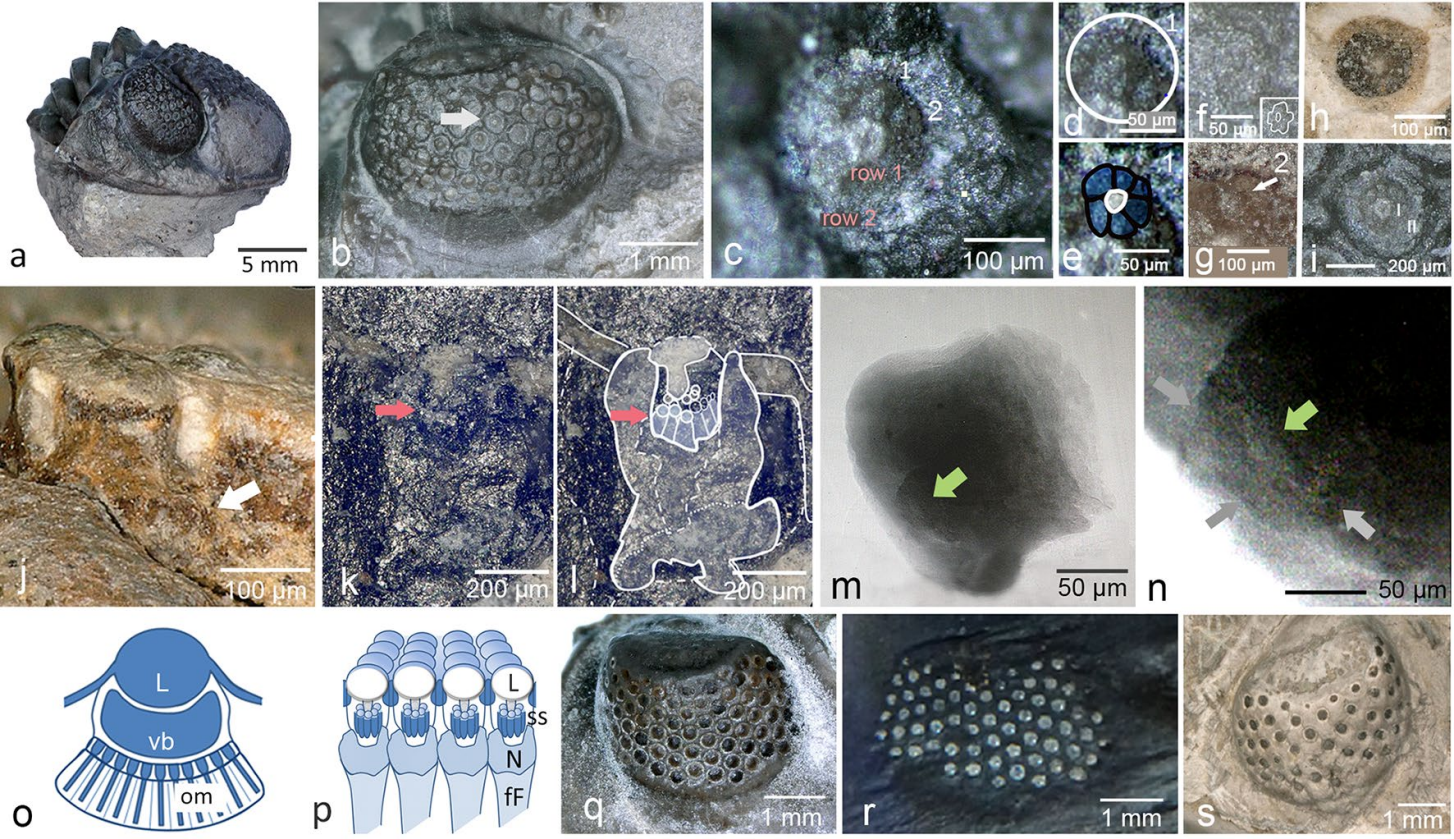
Structure of the visual unit of phacopid trilobites. (**a**) Cephalon of *Phacops imitator* Struve 1970. (**b**) Right eye of a, arrow: position of (**c**). (**c**) Ommaidial cross-section; 1,2 distinct ommatidia, see (**d**, **e**, **g**). (**d**, **e**, **g**) Cellular structure of ommatidium 1 and 2 of c, arrow indicates rhabdom). (**f**) Central element of the visual complex. (**h**) One ring of ommatidia in *Geesops schlotheimi* (Bronn, 1825), (**i**) two rings (I, II) of ommatidia in *P. imitator*. (**j**) *G. schlotheimi*, longitudinal aspect of the visual units’s complex. Arrow: neuropil (?). (**k**, **l**) Longitudinal section of visual complex (*P. imitator)*, arrow indictes lenses of the sub-systems (small ommatidia). (**m**) synchrotron radiation; visual capsule of *G. schlotheimi*, showing columnar elements (ommatidia), green arrow: position of (**n**). (**n**) Proximal side of ommatidium in (**m**). Rhabdom (pink arrow), and cell boundaries (grey arrows). (**o**) Schematic drawing of the eye of *Ampelisca*. (**p**) For comparison: schematic drawing of the internal structure of the hyper-compound eye of *Geesops schlotheimi* (Bronn, 1825), section. (**q**–**s**) Different phacopid schizochroal eyes with increasing distance between the lenses, probably because of progressive fusion of adjacent lens-systems. (**q**) *G. schlotheimi* (**r**) *C. ferdinandi* [under UV-light] (**s**) *Phacops latifrons* (BRONN, 1825), note the extremely wide distances between the lenses. fe ‘*fibers*', L lens, N neuropil, om ommatidia, ss sub-systems (small ommatidia), vb vitreous body.

Figure 3a shows this negative, Fig. 3b,c a section in a slightly processed version. It shows an X-ray radiograph of the compound eye of the *Phacops* (*Chotecops*) *fecundus* specimen WS 1135 2c. The ‘*fibers*' show up as bright lines or striae of irregular width, curving somewhat distally. Below the red arrow, however, one can see a single ‘*fiber*' widening, its top covered by a u-shaped, sponge-like structure. On this sit, as in a nest, ~ 6 small columns (~ 180 µm high, ~ 70 to 80 µm wide), embraced distally by a thin unclear bright 'collar' (~ 25 µm high), and each of them is topped by a sphere (~ 70 to 80 µm wide). Obviously, the large, all-covering lens above the complete unit is missing here as in the other adjacent elements. In our view, in principle, what we see here appears, at least in part, to repeat a design which we had previously found in an X-ray tomography of the eye of another phacopid trilobite, *Geesops schlotheimi* (Bronn 1825)^1,23^. In this, we also find a u-shaped ‘nest' hanging below a dense unit (Fig. 3e), here still covered by the large oval lens and a row of smaller spherical units below. We may interpret the small spheres in Fig. 3b–d as small lenses, sitting on top of tiny individual receptor units, together forming small ommatidia. We appreciate that any interpretation of these unusual internal structures of the visual unit is based on the very limited data provided by a single element alone. This hypothesis is, however, further confirmed by a synchrotron analysis showing a ‘corona' of small spheres of same density as the main lens atop of them (pink arrow, Fig. 3i). Even below the spheres, a kind of small bright collar can be identified (Fig. 3b,c), perhaps representing relics of the (reduced) crystalline cones^1,24–26^. More evidence that the tiny columns are relics of ommatidia is given by cross sections of visual units of different phacopid species. They show the typical structures of ommatidia in apposition compound eyes: ~ 6 relics of receptor cells grouped rosette-like around the fossil relic of a central rhabdom (Figs. 3h, 4c–i, see discussion also). These small ommatidia are situated on this sponge-like ‘nest', which may be interpreted as a neural network, the first level of processing of the visual input of this unit (Figs. 1r, 2j,p, 3b,c). The same outer complex, surely containing the receptor units, connected to ‘*fibers*' we find in a thin section of the Moroccan phacopid trilobite *Phacops tafilaltensis* Alberti, 1983 (Fig. 1r), and its contents in the synchrotron analysis of another phacopid trilobite *Barrandeops cf. granulops* Chatterton et al.^23^ (Fig. 3f,g).

In summary, we find the ‘*fibers'* ending distally in a u-shaped foamy ‘nest' (neuropil), a capsule containing the putative receptor units that in *C. fecundus* and other phacopid trilobites show up as a group of small, vertical columns, covered by a small row of spherical elements (probably the lenses of the small ommatidia, Fig. 3a–d), and finally the large lens above them all on top. They all build a single visual unit in the overall compound eye containing tens, in cases up to hundreds of these visual units, seen from outside as normal facets. Further on, we shall call these fossil relics of small ommatidia ‘subommatidia'.

To exploit the abilities and the newly developing modern techniques, Stürmer’s best known specimen, WS 295 (Fig. 2a,d), with which the discussion begun (now SNSBBSPG1930III8, Bayerische Staatssammlung Munich), was studied afresh by X-ray tomography at the Bayerische Staatssammlung Munich. Unfortunately, the extremely well preserved specimen WS 2617 (Fig. 1a) is lost and has survived only as a cast. The condition of this specimen (WS 295) frequently examined during the last almost 50 years had not improved and the delicate pyrite structures had decayed to a great extent. Some units, however, had deteriorated less than others and so allowed further insights, and importantly, this new analysis reveals fresh details of the underlying neuronal networks. Figure 2 shows that the whole compound eye consists of a kind of capsule. It protrudes into the interior of the cephalon, ending in a kind of tube (white arrow Fig. 2b). A similar capsule can be observed in another specimen of *Asteropyge* (WS 2236, black arrow Fig. 2f). The distal sensory units below the lenses in this specimen are represented, probably due to reflections, as bright triangles (Fig. 2b,c,e). The adjacent ‘*fibers*' can be made out vaguely, but some of them are still perceptible. In *C. ferdinandi,* they terminate on a layer of globular elements (Fig. 2c, compare structures indicated by pink arrows in Fig. 1), followed distally by the pyramidal structure (Fig. 2e, red arrows in Fig. 1m,q). The latter consists of layers of spherical elements, increasing in number but decreasing in size (Fig. 2e, comp. 1q also). This suggests a hierarchical arrangement of neuronal processing of the visual inputs of the systems, enhancing the processing of information step by step, perhaps in a sense of pooling.

## Discussion

Compound eyes, a plesiomorphic character of arthropods, consist of repeated identical units, the ommatidia, from outside seen as facets. Basic so-called apposition eyes have been found in a trilobite of the lower Cambrian, and thus this principle of vision is at least more than half a billion years old^1^. Recently proper apposition compound eyes were confirmed in the Silurian trilobite *Aulacopleura koninckii* (Barrande, 1846)^25^. Such eyes are still are still present in many diurnal insects and crustaceans living today. The apposition compound eye corresponds to the holochroal eye of trilobites, and consists of often numerous identical units, the ommatidia (Fig. 1e). Each ommatidium consists of a dioptric apparatus, focusing the incident light onto a light-guiding structure, the rhabdom. The latter is part of the receptor cells, which contain the visual pigments. The energy of the light changes the sterical configuration of these pigments, generating a low electrical signal, processed by the nervous system. In the simplest forms of apposition compound eyes all colours and contrasts in the visual field of an individual ommatidium in its simplest form are summed up to one signal, and the total picture seen is mosaic-like^10,27^. Among other factors, the acuity of an apposition compound eye depends on the number of facets, and the angle of acceptance of the rhabdom. Apposition compound eyes still are the typical visual system of diurnal arthropods, such as insects and crustaceans.

There had been prognostic indications that the visual systems of phacopid trilobites, though in appearance true compound eyes, were not typical apposition eyes, as holochroal eyes obviously are. In the nineties, Fordyce and Cronin^19,20^ investigated trilobite eyes, and applied mathematical approaches to work out how the compound eyes of trilobites sampled their visual world. The product of the facet diameter (*D*) and the interommatidial angle (*Δφ*) gives the value of the Eye Parameter: *DΔφ* for squared array of facets, D (√3/2) Δφ for hexagonal arrays of facets of apposition compound eyes, a value which is a reliable indicator of the photic conditions in which the arthropod lived^28–32^. For modern arthropods with normal apposition eyes, this eye parameter ranges from ~ 0.1 for diurnal organisms, active under bright light, and ~ 20 for crepuscular, nocturnal or deep sea arthropods. For the schizochroal eyes of *Eldredgeops* (*Phacops*) *rana crassituberculata* and *Eldredgeops* (*Phacops*) *rana milleri,* the authors found eye parameters between 10 and > 150, greater than in any living arthropod. They also investigated holochroal eyes of different trilobites (*Asaphus cornutus*, *Isotelus gigas*, *and Homotelus* sp.), and found eye parameters comparable to those of modern nocturnal organisms. They concluded that each ommatidium of the schizochroal eye probably served as a miniature lens eye^19^, conforming with the ideas of authors mentioned earlier, who saw a small retina in the capsule below each of the large lenses^13–21^, and that these eyes were equipped with multiply overlapping visual fields. These results may suggest that the schizochroal eyes had a functional system that could not be characterised by the eye parameter, because their system is very different from the apposition type.

There was a second aspect also. By zoologists, and opticians, the so-called *f*-number (*N* = *f*/*D*, *f* focal length, *D* diameter of the lens) often is used to characterise a visual or camera system, describing the effectiveness of light capturing. While due to the large lenses low *f*-numbers are common in single-chamber eyes, in normal apposition eyes the *f*-number normally lies at about *N ≈* 2^33–36^, the *f*-number in phacopid trilobites lies at *N ≈* 1.1^37^. In the holochroal eye from the base of the early Cambrian (Cambrian Stage 3, ~ 521 to 523 mya) trilobite *Schmidtiellus reetae* indeed was *N ≈* 2.2^1^, so the *f*-numbers in phacopid eyes are low compared to those for apposition eyes, suggesting that they are probably not of apposition or a modified type.

As we have seen the conception that the phacopid eye consisted of many lenses, each with a capsule below, floored by a small retina and thus structurally similar to a camera eye of vertebrates or cephalopods was not entirely correct. Instead, the puzzling eye of the phacopid trilobites rather probably is an aggregated hyper-compound eye, where numerous ommatidia lie underneath a common large lens, forming a small compound eye under each of the large lenses, and there are several tens to hundreds of such systems forming one lateral phacopid eye. There is just one example among living arthropods equipped with a similar system—the amphipod *Ampelisca callopia*^38^, (Fig. 2o). These crustaceans are tube dwellers, and can be found in sediment at moderate depths^39^, probably not too dissimilar to the life habitat of many phacopids. In these and some other amphipods, the ommatidia form a small retinula sharing one larger lens, and the system contains a fluid-filled space in between, functioning as a vitreous body^39–41^ (Fig. 4o). There is, however, commonly just a single eye or up to three separate ones at each side of the head, and so it does not form a hyper-compound eye as in phacopid trilobites.

### The ‘fibers'

There can be no doubt that the ‘*fibers*' actually exist, and that they are not preservational artefacts because they appear frequently, in different specimens, species and in various modes of preservation (*Chotecops* (*Phacops*) *ferdinandi* (Kayser, 1880), *Phacops tafilaltensis* Alberti 1983, and *Rhenops* (*Asteropyge*) sp. (Fig. 1a–c,f,g,k–r)). We showed that the fibres observed by Stürmer and Bergström^7^ are not gill filaments, because they connected to the visual units (1:1 relation, Fig. 1k-o,r); they widen proximally and do not taper, as the gill filaments which in cases underlie them, do. Nor are the ‘*fibers*' light guides, because they are not part of the dioptric apparatus; instead they follow the sensory system proximally (Fig. 1k-o, r).

### The sub-ommatidia

In Fig. 3a–d (WS 11352c), several ‘*fibers*' can be clearly seen, most of them obliquely abraded. One of them, however, is crowned by a u-shaped area with a fluffy appearance, topped by about six short rods, each capped by a sphere of approximately the same diameter, and showing a thin bright layer in between. Because it echoes the entire constructional principle of any typical (apposition) compound eye, this suggests an interpretation, that each of these elements is actually a small, stretched ommatidium. Cross-sections of other specimens indeed show the typical structure of about 6 elements (relics of receptor cells). Circularly arranged around a central element (Fig. 4f) they show distinctly a bright centre, each corresponding to the relics of a central rhabdom, encompassed by the receptor cells (Fig. 4d,e,g,h). In some cases, even the membranous boundaries of the relicts of cells can be identified (Fig. 4c,d,e,g,m,n). The meaning of the bright central structure of the entire complex (Figs. 3h, 4c,f,i) remains unknown so far. It vertically continues from the lens (Fig. 4k,l) and protrudes into the centre of the system. It is remarkable that in *G. schlotheimi* (Fig. 4h), *B. cf. granulops* (Fig. 3f,g), and *C. ferdinandi* (Fig. 3b,c), there exists just one ring of sub-ommatidia, and *P. imitator* (Fig. 4a) has two of them (Figs. 3h, 4c,k,l). Additional evidence of the presence of ommatidia in the capsule of the visual units of phacopid trilobites is given by a synchrotron analysis of such a capsule of *G. schlotheimi*. Inside the capsule, we see several columns, one even with a central bright rhabdom, surrounded by receptor cells; their boundaries, though indistinctly, are visible (Fig. 4m,n).

The relative distances between the lenses in the visual surface are wider in some phacopid trilobites (*P. imitator*, *P. latifrons*) than in *Chotecops* and *Geesops* (Fig. 4a,b,q–s). This supports well the model of their development as explained later in the supplement (S1).

They all reside on a u-shaped tissue, also shown in different species: *Barrandeops granulops* Chatterton et al. 2006 (Fig. 3f,g), *Geesops schlotheimi* (Bronn, 1825 (Figs. 3e, 4j), and *Phacops tafilatelensis* Alberti 1983 (Fig. 1r). We interpret the latter as a neuropil, probably processing the inputs of the small ommatidia. This continues distally as the ‘*fibers*' discussed here. These ‘*fibers*', originally observed by Lehmann^6^, Stürmer and Bergström^7^ consequently may be interpreted as the fossil relicts of the efferent nerve of this complex visual unit, combining all information of the small ommatidial units, and leading them to the central nervous system. In some specimens, one can observe how finally the fibres converge to central structures, deep within the head, and outside the eye (pink and red arrows in Fig. 1c,m,n,q,p), which must be further processing neuropils.

These observations give rise to a new interpretation of the results we presented earlier^23^ in the phacopid trilobite *Barrandeops cf. granulops* Chatterton et al*.* 2006. Our previous analysis using synchrotron radiation is consistent with that reported here. There, we see as in Fig. 3f,g vertical columns, each topped by a spherical structure. We interpreted these as receptor cells, but since they are ~ 80 µm in diameter (approximately of the same diameter as the columns described here), they seemed remarkably large (receptor cells: normally ~ 2 to 10 µm in living arthropods^39^). We thought that the rounded structures reported in 2013 were visible parts of columns lying behind, but it is now evident to us that the columns are the sensory units of the ommatidia, and that the spherical structures are their lenses. Finally, we also find the u-shaped supposed neuropile below, described in 2013 as having a fluffy cotton-like appearance (white arrow Fig. 3f,g).

To summarize, in the seventies, Wilhelm Stürmer found filamentous structures, highly controversial as discussed at that time, and not explained until he died and many decades later. This led, however, to the discovery made here, which allows to speculate that the schizochroal eyes of phacopid trilobites are complex visual systems—hyper-compound eyes, built of ~ 6 small sub-ommatidia grouped around a central axis below each of the large lenses of the typical schizochroal eye (Fig. 4p). Some phacopids (e.g. *P. latifrons*) seem to possess at least two rows of ommatidia. The whole lateral eye of a phacopid trilobite comprises in some cases of about 200 sub-compound-eyes. This provides the potential of a highly sophisticated system leading to further processing of the visual inputs, such as pooling, superpositioning, contrast enhancements, sharing of functions between the sub-ommatidia (e.g. for colour discrimination), and other uses of available information. These may be organised by a neuropil below and a more proximal neuronal centre. This special and unique form of a hyper-compound eye may be an adaptation to dim light conditions, because all subsystems capture light through one large lens.

The highly discussed ‘*fibers*' Stürmer and Lehmann had discovered indeed are integral elements of this system, they are obviously the fossil relicts of efferent nerves of each sub-compound eye as Lehman had supposed correctly, and lead to the more proximal neural centre mentioned, in contrast with the common perceptions of their time.

## Materials and methods

Material (Specimens) *Asteropyge* (*Rhenops*) sp., Kaub Formation, Hunsrück Slate Group, Lower Siegenian/Emsian Stage, Lower Devonian; Bundenbach area, Hunsrück, Germany, [WS 2234, WS 2882]; *Barrandeops cf. granulops* Chatterton et al*.* 2006, Upper Emsian, lower Devonian, Hamar Laghdad, Morocco. *Chotecops* (*Phacops*) *ferdinandi* (Kayser, 1880), Kaub Formation, Hunsrück Slate Group, Lower Siegenian/Emsian Stage, Lower Devonian; Bundenbach area, Hunsrück, Germany, [WS 4.1/503, WS 295, WS 609, WS 609(3), WS 822, WS 1832, WS 2617, not numbered specimen] ; *Cyclopyge sibilla* Šnajdr, 1982. Ktaoua Fm., Upper Ordovician, El Kaid Errami, Morocco; *Geesops schlotheimi* (Bronn, 1825), Ahrdorf Formation, Eifelian, Middle Devonian, Gees/Gerolstein, Eifel, Germany; Hollardops merocristata Le Maître,1952, Middle Devonian, Maider, Jbel Issoumour, SE Morocco; *Paladin eichwaldi shunnerensis* (King 1914), Millstone Grit-Fm., Namurian, Upper Carboniferous, Great Shunnar Fell, Yorkshire, UK; *Pedinopariops brongniarti* (Steininger, 1831). Ahrdorf Fm., Flesten Mb., Gees/Gerolstein, Middle Devonian, Eifel, Germany; *Phacops imitator* Struve, 1970*,* Nohn Fm. Emsian, lower Middle Devonian, Üxheim, Eifel, Germany; *Phacops tafilatelensis* Alberti 1983, Emsian, Timrhanrhart Fm., Lower Devonian, Jbel Gara el Zguilma, Morocco; *Gerastos cuvieri* (Steininger, 1831) Ahrdorf Formation, Eifelian, Middle Devonian, Gees/Gerolstein, Eifel, Germany; *Pricyclopyge bindosa* (Barrande, 1872), Czech Geological Survey, CGS XB 139 Šárka Fm, Darriwilian, Middle Ordovician, Prague, Czech Republic.

Our figures are own photographs made of the radiographs: SCB = Collection Christoph Bartels, Bochum/Steinmann Institute, University of Bonn, except those from Stürmer et al. (1980, pl. iV, Fig. 15, 15a) [here: Fig. 1a-c] and Stürmer and Bergström (1973, pl. 21 b,c) [here: Fig. 1n,q] as mentioned in the text. Light microscopy: Keyence digital-microscope (VHX-700F, VHZ-00R, VHZ-100R), University of Cologne,Institute of Biology Education (Zoology); Fig. 3e GE phoenix|X-ray, v|tome|x micro CT) at the Steinmann Institute, University of Bonn, providing a voxel size of 38–82 μm, unfiltered projections, at 170 kV and 180 *μ*A.; synchrotron: ESRF Grenoble ID19 (2010).

Figure 2b,c,e; S1 Bavarian Natural History Collections (SNSB) facilities, phoenix|X-ray nanotom m (phoenix_X-ray, GE Sensing and Inspection Technologies GmbH, Wunstorf, Germany). RoessnerG_SNSBBSPG1930III8_b, Voxel size 35.1 µm, Image number 1400, Voltage 140 kV, Current 0.07 mA, Filter 0.2 mm Cu.

## Supplementary Information

Supplementary Information 1.

Supplementary Information 2.
