# Supplementary material for: A 390 million-year-old hyper-compound eye in Devonian phacopid trilobites
Source: Sci Rep. 2021 Sep 30;11:19505. doi: 10.1038/s41598-021-98740-z (PMC8484558; doi:10.1038/s41598-021-98740-z)
Supplement: Supplementary file 1 — Supplementary Information 1. [file 41598_2021_98740_MOESM1_ESM.docx]

**Hypothesis about a possible evolutionary way to develop a hyper-compound eye out of a regular apposition eye (holochroal eye) and its assignment to phylogenetic contexts of the evolution of compound eyes**

**Development of schizochroal eyes and comparison with recent analogues**

There are two theories that have been proposed for the origin of the schizochroal eye. The first is that it arose by paedomorphosis from the early stages in the larval development of a holochroal eye (Fig. S1 I,m)^1-3^. This is well supported by what is known of the juvenile stages of development of the holochroal eye of the Carboniferous proetid *Paladin*^4^, and also of the upper Cambrian olenid *Olenus*^5^. In both of these trilobites the juvenile lenses are very large relative to the dimensions of the visual surface, and they are separated by a kind of cuticular 'wall' (interlensar sclera). The schizochroal eyes may have retained this organisation, simply by enlarging the individual visual units , thereby capturing more photons per unit, while the holochroal eyes, in contrast, retained the original structure in the adult, but sometimes enlarging it to become high-resolution compound eyes with several thousands of lenses in each eye. An alternative hypothesis is that schizochroal eyes originated by coalescence of the small lenses of holochroal eyes resulting in a smaller number of larger units (Fig. S1 n-t). These two hypotheses are not mutually exclusive, if we agree to assume that there may be two genetic programmes: the first installing small, complete larval ommatidia, and a second which orders them – either adjacent to each other (Plan A), or collecting nearest neighbours below one enhanced central element (Plan B), (Fig. S1 n-t). The geometry even allows hyper-systems as shown in Fig. S1 u. The third concept is that of a small retina flooring the capsule of the schizochroal eye, as was suggested by several authors^1, 6-12^, but as we have seen before this seems not to be the case.

The large interspaces between the lenses in schizochroal eyes of phacopid trilobites are striking (Fig. 3q-s; S1 j-m), which makes them different from the densely packed lenses of most of the most common and basic eye of trilobites, the holochroal eye (Fig. S1 f-i)^3^, and indeed the eyes of most insects and crustaceans. Here there may be up to thousands of lenses (Cyclopygidae, Fig. S1 g,h), densely packed with only very small interspaces. The lenses of phacopid (schizochroal) compound eyes may be larger than 2 millimeters (*Drotops megalomanicus* Struve, 1990) and also in the other phacopids the lenses are remarkably larger than in most other trilobites, the cuticular 'walls' in between, the interlensar sclera being approximately half as wide as the lens (Fig. 3q-s; S1 j)^3^. It is perfectly possible that during the growth and development of schizochroal eyes groups of, say, six neighbouring lenses migrated, each under a centrally positioned single lens, which grew larger than usual and covered them all. Its central structure obviously developed a special form (round to star-shaped element (Fig. 2h; 3c,f). It seems evident that the number of lenses visible from the outside, developing in the visual surface of schizochroal eyes is not as high as in the holochroal eyes, probably, because those which would otherwise be positioned in the later interspaces between the lenses, undertake this kind of collective migration (Fig. S1 n-v). This migration may have taken place most simply in such species as *G. schlotheimi*, *B. granulops* and *C. ferdinandi*, which have one row of ommatidia around the centre (Fig. 2 b,c; f,g, 3h,m,n), whereas two such rows are evident in *P. imitator* (Fig. 2h, 3c,k,l). There may have been even more of them, such as *P. latifrons,* because of the even wider interspaces between the individual lenses (Fig. 3s).This idea is strengthened by the fact that the interspaces between the lenses seen from outside are larger in the latter (Fig 3q-s), because more ommatidia, originally situated in these spaces, now sit below the large lens. There are examples of phacopid eyes, where this migration of smaller units seems to be still on its way, when rows of small lenselets lie between the larger ones (Fig. S1 s). The physical advantage is that the small ommatidia share the wider aperture of the large lens, and possibly in such a complex system any differentiated work-sharing, such as colour vision (UV discrimination, discrimination of polarised light by some of the ommatidia), neuronal superposition, and other properties could develop and may have become very effective. Perhaps even some mirror optics may have been present in the capsule containing the sensory system – we probably never shall know.

In the oldest trilobite compound eye described so far , from the base of the Lower Cambrian^1^ the ommatidia remain very separate from each other and are embedded within a wide capsule. From here two evolutionary pathways can be envisaged, the first being that when the resolution is increased (which implies a larger number of lenses), the units were multiplied and squeezed^comp. 13,13^ to form a holochroal eye with numerous facets - or the ommatidia, multiplied when growing also, coalesce together to form a schizochroal eye, putatively increasing sensitivity, and perhaps even hiding a sophisticated visual system, conserving the original capsule as principle of construction.

***Comparison with recent forms***

The compound eye in phacopids is a kind of hyper-compound eye, because here the 'retina' consists of a series of individual small ommatidia, as can be shown here in *Chotecops (Phacops) ferdinandi* and other species of phacopid trilobites to be probably the case. There are several examples among living arthropods, with ocelli (single-lens units, each with a small retina below a large lens), which serve as the main eyes. Buschbeck and her group suggest that the larval stemmata, the eyes of holometabolous insects (single-chambered eyes) may have evolved from ommatidial compound eyes being integrated to one system^15^. These stemmata either could develop through the expansion of a single ommatidium, such as in the larvae of ant-lions (*Cicindela chinensis* Thunberg, 1781*,* Neuroptera^16^, tiger beetles (*Euroleon nostra* (Geoffroy in Fourcroy, 1785), Coleoptera^17^, dobsonflies (*Protohermes grandis* (Thunberg, 1781), Megaloptera^18^, or certain diving beetles, which possess as many as 6 ocelli on each side comprising a high number of ommatidia even forming a multilayered retina, probably providing a highly differentiated vision (*Thermonectus marmoratus* (Gray, 1831), Coleoptera^19^. Similarly the evolutionary development of the lateral eyes of spiders has been discussed^20-22^. Here more relevant probably for comparison are the stemmata which evolved by the fusion of many ommatidia – as in the larvae of the saw fly *Perga*^23^. All these stemmata, mentioned because of their optical properties, and the equivalent number of receptors are discussed as image forming^15^. In the schizochroal eye the number of receptors below each of the large lenses may be too low for any image formation, if each of the small ommatidia summed up the inputs as they normally do in apposition eyes. If, however, some superpositioning between the different units (corresponding to the individual facets with their systems) existed^24^, an image formation here also may be envisaged. If the sophisticated systems of the larval eyes of insects, especially as of *Perga*, originated from ommatidial compound eyes, the system of the phacopid eye may be based on a genetic program not very dissimilar to, or even being related to that of these insects, and the former idea of paedomorphosis again may be entertained. All recent organisms using such principles live under low light conditions or have, as in the case of the strepsiterian *Xenos peckii*^25^, very small eyes, thus have to enhance their capacities to capture photons effectively. So, in conclusion ,all these recent arthropods possess eyes composed of fused receptor units, but none of them forms a hyper-compound eye. Only in the schizochroal eye of phacopid trilobites do the subunits keep their individual ommatidial character: receptor cells surrounding a central rhabdom, each system covered by an individual lens.

The most relevant arthropod eye for comparison here is probably that of the marine amphipod *Ampelisca* (Fig. 3o). It is equipped with one to three pairs of single lens-eyes, which clearly derived by fusion of several ommatidia to one visual unit^26-29^, (Fig. 3o), as our model for the development of schizochroal eyes suggests also. In the amphipod ocellus the ommatidia are closely packed, situated under a common cuticular lens, with a vitreous body in between. The ampeliscid amphipodes (Crustacea) are tube dwellers that inhabit soft deposits, and live at moderate depths^27^. The large phacopid eyes thus may likewise have been adapted to low light intensities. This assumption is strengthened by the research of Barchert *et al.* in 1992^30^ about the Devonian faunas of the Kess-kess mountains of Morocco. They report that the absence of blind taxa and burrowers may indicate that the community lived within the photic zone on a hard substrate, sometimes exposed to slight currents. It is remarkable that no phacopids and asteropygines have been found yet in the Kess-kess mound environments, in contrast to the late Dajelan 'Red Fauna' from Hamar Laghdad, where they frequently occur^31,32^. The latter fauna uppermost Emsian, lower Devonian) is much younger that the Kess-kess fauna and lived on and in a lime-mud substrate. The milieu rather probably was subsident and deeper. Feist and Belka assume that the phacopid trilobites avoided reef-like environments with hard substrates and turbulent waters^32^. This is in accordance for example with findings of other phacopids, as such of the Middle Devonian Eifel fauna, where the phacopid trilobites always occur in fine grained, silty limestones. Thus the life-habitat of ampeliscid crustaceans of today and of phacopid trilobites may not have been too dissimilar, and may have, in principle, led convergently to similar adaptations of eye-structure. There is, however, no example among living arthropods, with ocelli formed by ommatidia. Only in phacopid trilobites do the subunits below the large lenses keep their individual, ommatidial character with receptor cells, surrounding a central rhabdom, and a covering individual lens - proper (hyper-)compound eyes serving as the main visual organs in phacopid trilobites.

**Neural structures, and correlation to the phylogenetic tree of arthropods**

A second aspect of the investigations presented here is the bearing they have on the optical part of the nervous system. The first component we find below the small ensemble of ommatidia, is the u-shaped layer of ~120 µm thickness and ~300 µm width, in the radiographs as well as in the thin sections showing a foamy appearance (Fig. 1r; 2a-d; 3j). It stands to reason here to presume, that here the first level of information coming from the set of small ommatidia is sorted out and processed, before this information is transported by Stürmer´s 'fibres' (the 'f-elements'), now interpreted as nerves, to the next level. In the figures 1c,e,n,p,q; 4c,e we see these 'fibres' passing a kind of plate consisting of small globular elements, then fusing to a hierarchically organised system, from numerous large cubes to fewer ones (Fig. 1 n,q, 2e), with a thin filament finally leaving the more or less pyramidal organ (Fig. 1m, 4e).

The fine-structure of the neuropil mentioned, which lies below the capsule, may suggest the interpretation of Fig. S1 a,b. If we accept the interpretation that each visual unit of a phacopid compound eye is a small compound eye itself, we can observe in Fig. S1 a,b that outside the facet´s capsule lies a thin (~50 µm) lamina (Fig. S1 a-c). When compared to other arthropods this very probably is the typical position for a neuropile, comparable to the so-called lamina, a neuronal layer, where the first level processing takes place, for example, temporal differentiation of the signals, lateral inhibition (contrast enhancing) or neural superpositioning. It is closely attached proximally to a flattened element (~50 µm thick), perhaps a fossil relic of a neuropile comparable to a medulla, which conceivably took over tasks such as colour discrimination, elementary motion detection (see below) and intensity coding. There is no constriction between these two elements – probably indicating the absence of a chiasma (the distance between these both lobes probably would have been larger, if there was a chiasma). If so, the image perceived at this level was upright rather than inverted, but this must remain uncertain. The last element visible probably is a part of the 'f-filament', appearing again very bright in the x-ray photographs. Whether a third neuropile (Lobula) exists, cannot be established – probably it does not. One has to remember that this sequence of structures lies below the visual unit of an individual large lens. As we have seen, however, each unit is constructed as a small individual compound eye itself, and thus seems to repeat the probable concept of organisation of optical lobes of a typical apposition compound eye. Strausfeld and colleagues assumed that the sessile compound eyes of trilobites are a derived condition from the plesiomorphic stalked eyed configuration first resolved in radiodonts^33^, and the interpretation of the sequence of optical neuropils as given here fits perfectly into the sequence given by Strausfeld^33^, showing no chiasma, and no third and fourth optical neuropil (lobula and lobula plate). It is most similar to the arrangement of neuropils of Radiodonata or Xiphosura. That the neuropils in phacopid trilobites are attached to each other seems to lead towards to modern systems as such of that of the scutigeromorphs (Fig. S1 e).

Thus, in conclusion it has been shown that the 'fibres', ('f-elements') that Stürmer and Lehmann had described, really exist as part of the compound-eye-system and that they are not gill filaments. They are fossil relics of nerves that brought the information of the visual units below the wide phacopid lenses to the next level of neuronal processing, which lies still inside the compound eye´s capsule. The most interesting point here is that below each lens putatively is nested an array of several ommatidia. One may form a hypothesis that the generation of this pattern follows a genetic program of at least two steps. The first is to build separated units (ommatidia), as in every larval holochroal eye. Then the development may pass alternative ways: it simply may produce more such units during growth, finally forming a densely packed, ideally hexagonally close-packed holochroal compound eye. The second way starts from the same larval eye, with its separated lenses, but neighbouring units migrate under a central element, which widens, while the smaller ones remained covered. Thus in phacopid trilobites we have a 'hyper-'compound eye, which from the physiological point of view, has potential to increase sensitivity by sharing the wide aperture of one lens with several smaller systems, and perhaps to share functions such as colour vision, contrast- enhancing facilities, or simply pooling.

The study also has given insights into the neuronal background; there is a first centre of processing directly below the small ommatidia, followed by a hierarchically organised preliminary level inside the eye capsule, both connected by the 'f-elements' in question.

The eyes of phacopid trilobites reveal the sequence of Lamina and Medulla, typical for arthropods, but with no indication of any chiasma. There is a pyramidally-shaped centre to integrate the efferent infromation. Where the information of all units of the compound eye are processed, remains unrevealed, probably it lies in the outer regions of the brain (protocerebrum). Because further neuropils are missing, such as a lobula or lobular plate, we find, when comparing the system given by Strausfeld *et al.*^33^ to that of phacopids, that the said trilobites represent the typical arrangement of 'old' systems, such as of the Radiodonta, of Leanchoiliidae, Merostomata or Arachnidae. That both neuropiles lie so close together seems to be dissimilar to those, and leading the way towards that of the modern arthropods, especially the Scutigeromorpha.


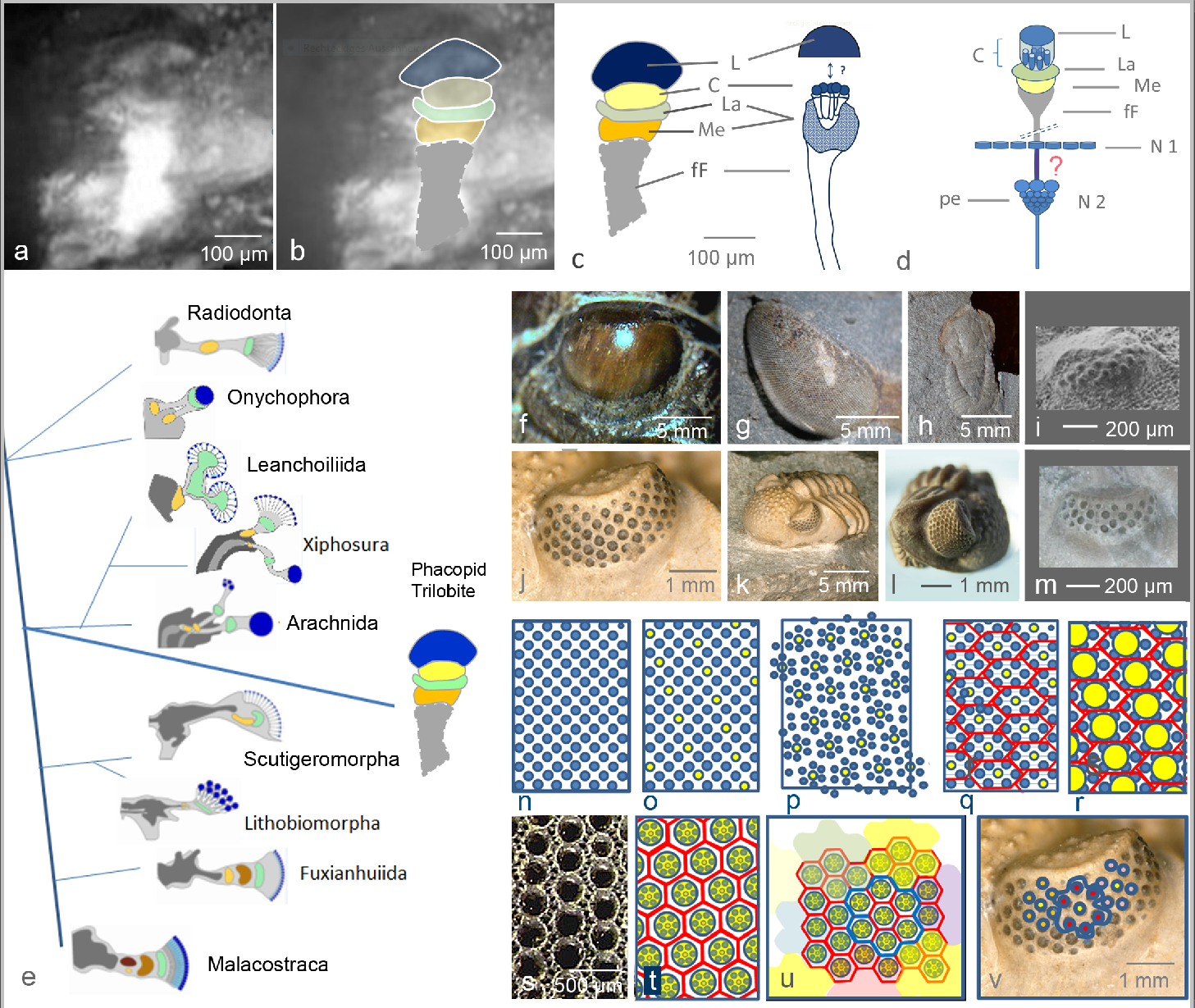


fe

fe

**Figure S1. | Assignment to the phylogenetic tree.** a) ct-scan of an individual visual capsule ('facet') of *Geesops schlotheimi* (Bronn, 1825), and interpretative colouring. b) Interpretation of a) and assignment to the findings before. c,d) Synopsis of the total system. e) Assignment of the neuronal organization to the phylogenetic context (phylogenetic tree after *Strausfeld et al.*^33, Fig. 11^). f) Holochroal eye of *Gerastos cuvieri* (Steininger, 1831), with smooth surface, several hundreds of facets invisible below. g) Right eye (exuvia), holochroal, of *Pricyclopyge binodosa* (Barrande, 1872), Czech Geological Survey, CGS XB 139, with several thousand facets. h) *Cyclopyge sibilla* Šnajdr, 1982. Note the hypertrophied compound eyes of this pelagic trilobite. i) Larval holochroal eye of *Paladin eichwaldi shunnerensis* (King 1914), Courtesy *Palaeontology*, Clarkson & Zhang, 1991^34^; note the interspaces between the facets. j) Schizochroal eye of *Geesops scholtheimi* (Bronn, 1825). k) Complete specimen of i. l) *Pedinopariops brongniarti* (Steininger, 1831) with schizochroal eyes. m) Larval state of the schizochroal eye of *G. schlotheimi*. Note the similarity to h. n-u) Model explaining the development of schizochroal eyes by consequent fusion of adjacent ommatidia: n) Initial situation, blue dots: ommatidia. o) Yellow dot: Prospective 'center of assemblage ' and covering lens. p) Assemblance of neighbouring ommatidia. q) Resulting honeycomb-like arrangement. r) Central large lens (yellow) expanding. s) Visual surface of *Hollardops merocristata* Le Maître 1952, note the lines of probable small lenses between the main rows. t) Final arrangement. u,v) Possible coupling to hyper-systems (Superposition possible). C capsule containing the sub-ommatidia, fe 'f-element', L ens, La lamina, M medulla, pe pyramidal element, n 1,2 neuropil 1,2.

References

1. Clarkson, E.N.K. The eye: morphology, function and evolution. in *Treatise on Invertebrate Palaeontology* [Part O- Arthropoda (Trilobitomorpha], (ed. R.C. Moore, R.C.) 14-132 (Geol. Soc. America and Univ. Kansas Press, 1997).

2. Clarkson, E.N.K. On the early schizochroal eyes of *Ormathops* (Trilobita, Zeliszkellinae). Mem. Bur. *Recherches Geol. Mineral*. **73**, 51-63 (1971).

3. Clarkson, E.N.K., Levi-Setti, R. & Horváth G. The eyes of trilobites: the oldest preserved visual system. *Arthropod Struct. Dev.* **35**, 247-259 (2006).

4. Clarkson, E.N.K. & Zhang, X.-g. Ontogeny of the Carboniferous trilobite *Paladin eichwaldi shunnerensis* (King, 1914). *Trans. Roy. Soc. Edinb. Earth Sci.* **82**, 277–295 (1991).

5. Clarkson, E.N.K. & Taylor, C.M. Ontogeny of the trilobite Olenus wahlenbergi Westergård, 1922 from the upper Cambrian Alum Shales of Andrarum, Skåne, Sweden. *Trans. Roy. Soc. Edinb.: Earth Sci.* **86**, 13-34 (1995).

6. Campbell, K.S.W. The functional anatomy of phacopid trilobites: musculature and eyes. *J. Proc. R. Soc. New South Wales* **108**, 168-188 (1975).

7. Bruton, D. & Haas, W. The anatomy and functional morphology of Phacops (Trilobita) from the Hunsrück Slate (Devonian). *Palaeontographica A* **253**, 29-75 (1999).

8. Clarkson, E.N.K. Fine-structure of the eye in two species of Phacops (Trilobita). *Palaeontology* **10**, 603-616 (1967).

9. Clarkson, E.N.K. The evolution of the eye in trilobites. *Fossils & Strata* **4**, 7–31 (1975).

18. Haack, S.C. The evolution and acuity of the schizochroal eye in trilobites. Evolutionary Theory 8, 69-72 (1987).

10. Fordyce, D. & Cronin T.W. Comparison of fossilized schizochroal compound eyes of phacopid trilobites with eyes of modern marine crustaceans and other arthropods. *J. Crust. Biol.* **9**, 554-569 (1989).

11. Fordyce, D. & Cronin T.W. Trilobite vision: a comparison of schizochroal and holochroal eyes with the compound eyes of modern arthropods. *Paleobiology* **19**, 288-303 (1993).

12. Bruton, D.L. & Haas, W. The puzzling eye of *Phacops.* *Special Papers in Palaeontology* **70**, 349–361 (2003).

13. Gehring, W.J. Chance and Necessity in Eye Evolution. *Genome Biology and Evolution* **3**, 1053-66 (2011)

14. Gehring, W.J., Kloter, U. & Suga, H. Evolution of the Hox gene complex from an evolutionary ground state. *Curr.Top. Dev. Biol.* **88**, 35-61 (2009).

15. Buschbeck E.K. Escaping compound eye ancestry: the evolution of single-chamber eyes in holometabolous larvae. *J. Exp. Biol.* **217**, 2818-2824 (2014).

16. Toh, J. & Okamura J.Y. Morphological and optical properties of the corneal lens and retinal structure in the posterior large stemma of the tiger beetle larva. *Vision Res*. **47**, 1756-1768 (2007)

17. Paulus H. Comparative morphology of the larval eyes of Neuropteroidea in *Recent Research in Neuropterology* (eds. Gepp, H., Aspöck, H. & Hölzel, H.) 157-164 (Proceedings of the 2nd Symposium Neuropterology, Hamburg, Graz, 1986).

18. Yamamoto, K. & Toh, Y. Fine-structure of lateral-ocellus of dobsonfly larva. *J. Morphol*. **146**, 415-429 (1975).

19. Mandapaka, K., Morgan, R.C. & Buschbeck E.K. Twenty-eight retinas but only twelve eyes: an anatomical analysis of the larval visual system of the diving beetle *Thermonectus marmoratus* (Coleoptera: Dytiscidae). *J. Comp. Neurol.* **497**, 166-181 (2006).

20. Land, M. F. Crustacea in *Photoreception and Vision in Invertebrates*. M.A. Ali, ed. (Springer, Berlin, 1984), pp. 401–438.

21. Paulus, H. F. Eye structure and the monophyly of the arthropod eye In *Arthropod phylogeny,* A.P. Gupta ed. (van Nostrand, 1979), pp. 299-383.

22. Morehouse, N.L., Buschbeck, E.K., Zurek, D.B., Steck, M. & Porter M.Molecular Evolution of Spider Vision: New Opportunities, Familiar Players. *Biol Bull.* **233**, 21-38 (2017).

23. Meyer-Rochow, V. B. Structure and function of the larval eye of the sawfly, Perga. *J. Insect Physiol.* **20**, 1565-1591 (1974).

24. Schoenemann, B. Trilobite eyes and a new type of neural superposition eye in an ancient system. *Palaeontographica* A **281**, 63-91 (2007).

25. Buschbeck, E.K., Ehmer, B. & Hoy, R. Chunk versus point sampling: visual imaging in a small insect. *Science* 286: 1178–1180 (1999).

26. Strauss, E. Das Gammaridenauge. [The eye of gammarid crustaceans.] *Wiss.* *Ergebn. dt. Tiefsee-Exped. Valdivia*. **20**, 1–84 (1926).

27. Elofsson, R., Hallberg, E. & Nilsson, H.L. The juxtaposed compound eye and organ of *Bellonci* in *Haploops tubicola* (Crustacea: Amphipoda)—The fine structure of the organ of *Bellonci.* *Zoomorph.* **96**, 255-262 (1980).

28. Nilsson, D.-E. Optics and Evolution of the Compound Eye in *Facets of Vision*. G. Doekele, R.C.H. Stavenga, eds. (Springer, 1979), pp. 30-73.

29. Hallberg, E., Nilsson, H.L. & Elofsson, R. Classification of amphipod compound eyes—the fine structure of the ommatidial units (Crustacea, Amphipoda). *Zoomorph.* **94**, 279-306 (1980).

30. Brachert, T.C., Buggisch, W., Flügel, E., Hüssner, H.M., Joachimski, M.M., Tourneur, F. & Walliser, O.H. Controls of mud mound formation: the Early Devonian Kess-Kess carbonates of the Hamar laghdad, Antiatlas, Morocco. *Geol. Rundschau* **81**, 15-44 (1992).

31. Crônier, C., Oudot, M., Klug, C. & De Baets, K. Trilobites from the Red Fauna (latest Emsian, Devonian) of Hamar Laghdad, Morocco and their biodiversity. *N. Jb. Geol.* **290**, 241-276 (2018).

32. Feist, R. & Belka, Z. Late Emsian (Devonian) trilobite communities from the Kess-Kess mounds, Hamar Laghdad (Anti-Atlas, Morocco). *N. Jb. Geol* **290**, 277-290 (2018).

33. Strausfeld, N.J., Ma, X.-o., Edgecombe, G.D., Fortey, R.A., Land, M.F., Liu, Y., Cong, P.-y. & Hou, X.-g. Arthropod eyes: The early Cambrian fossil record and divergent evolution of visual systems. *Arthropod Struct. Dev*. **45**, 152-172 (2016).

34. Clarkson, E.N.K. & Zhang, X.-g. Ontogeny of the Carboniferous trilobite *Paladin eichwaldi shunnerensis* (King, 1914). *Trans. Roy. Soc. Edinb. Earth Sci.* **82**, 277–295 (1991).
