# Supplementary material for: A 390 million-year-old hyper-compound eye in Devonian phacopid trilobites
Source: Sci Rep. 2021 Sep 30;11:19505. doi: 10.1038/s41598-021-98740-z (PMC8484558; doi:10.1038/s41598-021-98740-z)
Supplement: Supplementary file 2 — Supplementary Information 2. [file 41598_2021_98740_MOESM2_ESM.docx]

**'F-elements' in the phacopid trilobite *Chotecops* sp. from the Hunsrück Slate.**

Just the most expressive ones are used in this study.

Abbrevations: GPIBo = Steinmann Institute of Geology, Mineralogy, and Palaeontology, University of Bonn; HM = Hunsrück-Museum, Simmern; SCB/GPIBo = Collection Christoph Bartels, Bochum/ GPIBo; XRCB X-ray archive Christoph Bartels/ GPIBo; SMF = Senckenberg Research Institute and Museum of Natural History, Frankfurt/Main; SESAM Collection Management System of the Senckenberg Museum, database; SNSB-BSPG = Bavarian Natural History Collections (Staatliche Naturwissenschaftliche Sammlungen Bayerns - Bayerische Staatssammlung für Paläontologie und Geologie), Munich; WS = Wilhelm Stürmer; CWS Collection Wouter Südkamp (Hunsrück Museum, Simmern).

| X-ray | Repository of specimens | References | Comment |
| --- | --- | --- | --- |
|  |  |  |  |
| WS 295 | XRCB  SNSB-BSPG 1930 III 8 | Broili (1930): fig. 2/Plate 22, fig. 1  Broili, F. (1930) Weitere Funde von Trilobiten mit Gliedmaßen aus dem rheinischen Unterdevon. [Further findings of trilobites with appendages of the Rhinish Lower devonian]. *N. Jb. Miner. Geol. Paläont. B* **64**, 293-306 (1930).  Stürmer (1970): fig. 3  Stürmer, W. Soft parts of cephalopods and trilobites: some surprising results of X-ray examinations of Devonian slates. *Science*, *170*(3964), 1300-1302 (1970).  Stürmer et al. (1980): table 5, fig. 16  Stürmer, W., Schaarschmidt, F. & Mittmeyer, H. G. *Versteinertes Leben im Röntgenlicht*. [Petrified life under x-ray-light.], Kramer Verlag (1980).  Stürmer & Bergström (1973): plates 16e, 17a, b, 21b  Stürmer, W. & Bergström, J. New discoveries on trilobites by X-rays. *Paläont. Z.* **47**, 104-141 (1973).  Bruton & Haas (1999): figs. 19g-h/plates 12a, b, 13, 14  (Using the collection of Prof. W. Blind)  Bruton, D., & Haas, W. The anatomy and functional morphology of Phacops (Trilobita) from the Hunsrück Slate (Devonian). *Palaeontographica Abteilung A*, 29-75 (1999).  This research: Fig. 1n | Classical specimen  Lateral view |
| WS 609 | XRCB  GPIBo Edz 1 | Stürmer (1970): fig. 4b  Stürmer, W. Soft parts of cephalopods and trilobites: some surprising results of X-ray examinations of Devonian slates. *Science*, *170*(3964), 1300-1302 (1970).  Stürmer & Bergström (1973): plates 16f, 21a  Stürmer, W. & Bergström, J. New discoveries on trilobites by X-rays. *Paläont. Z.* **47**, 104-141 (1973).  Bruton & Haas (1999): plate 1a-c  (Using the collection of Prof. W. Blind)  Bruton, D., & Haas, W. The anatomy and functional morphology of Phacops (Trilobita) from the Hunsrück Slate (Devonian). *Palaeontographica Abteilung A*, 29-75 (1999).  This research: Fig. 1o,p |  |
| WS 613 | GPIBo Egr 477 | Stürmer (1970): fig. 4a  Stürmer, W. Soft parts of cephalopods and trilobites: some surprising results of X-ray examinations of Devonian slates. *Science*, *170*(3964), 1300-1302 (1970).  Stürmer (1974): fig. 3  Stürmer, W. (1974): Röntgenstrahlung und Paläontologie. [x-radiation and Palaeontology]. *Electromedica* **2**, 43-46 (Siemens AG, Erlangen, 1974).  Stürmer et al. (1980): table 3, fig. 9  Stürmer, W., Schaarschmidt, F. & Mittmeyer, H. G. *Versteinertes Leben im Röntgenlicht*. [Petrified life under x-ray-light.], Kramer Verlag (1980).  Stürmer & Bergström (1973): plates 18, 19a, 21d  Stürmer, W. & Bergström, J. New discoveries on trilobites by X-rays. *Paläont. Z.* **47**, 104-141 (1973).  Bruton & Haas (1999): fig. 2a-c  (Using the collection of Prof. W. Blind)  Bruton, D., & Haas, W. The anatomy and functional morphology of Phacops (Trilobita) from the Hunsrück Slate (Devonian). *Palaeontographica Abteilung A*, 29-75 (1999). |  |
| WS 673 |  | No figure by Stürmer & Bergström (1973) published  Stürmer, W. & Bergström, J. New discoveries on trilobites by X-rays. *Paläont. Z.* **47**, 104-141 (1973). |  |
| WS 822 | XRCB | This research: Fig. 1h-j |  |
| WS 873 |  | Stürmer & Bergström (1973): plate 21e  Stürmer, W. & Bergström, J. New discoveries on trilobites by X-rays. *Paläont. Z.* **47**, 104-141 (1973). |  |
| WS 1234 | SMF X288q  SMF-HS 612 | Stürmer & Bergström (1973): plate 16i  Stürmer, W. & Bergström, J. New discoveries on trilobites by X-rays. *Paläont. Z.* **47**, 104-141 (1973).  Bruton & Haas (1999): fig. 3a-c  (Using the collection of Prof. W. Blind)  Bruton, D., & Haas, W. The anatomy and functional morphology of Phacops (Trilobita) from the Hunsrück Slate (Devonian). *Palaeontographica Abteilung A*, 29-75 (1999). |  |
| WS 1832 | XRCB | Stürmer & Bergström (1973): plates 16c, 21c  Stürmer, W. & Bergström, J. New discoveries on trilobites by X-rays. *Paläont. Z.* **47**, 104-141 (1973).  This research: Fig. 1q |  |
| WS 2305 | XRCB  SMF-HS 575 (Cast) | Stürmer & Bergström (1973): plate 16b  Stürmer, W. & Bergström, J. New discoveries on trilobites by X-rays. *Paläont. Z.* **47**, 104-141 (1973). |  |
| WS 2482 |  | Stürmer & Bergström (1973): plates 16h, 21f  Stürmer, W. & Bergström, J. New discoveries on trilobites by X-rays. *Paläont. Z.* **47**, 104-141 (1973). |  |
| WS 2617 | SMF-HS 564 (Cast)  Original spcimen is lost | Stürmer et al. (1980): fig. 5/Table 4, fig. 15, 15a  Stürmer, W., Schaarschmidt, F. & Mittmeyer, H. G. *Versteinertes Leben im Röntgenlicht*. [Petrified life under x-ray-light.], Kramer Verlag (1980).  This research: Fig. 1a-c | Lateral view. Not studied by Bruton & Haas (1999) |
| WS 2882 | XRCB | This research: Fig. 1f,g |  |
| WS 4171 | SMF-HS 627  (Cast) | Bartels & Brassel (1990): fig. 78  Bartels, C., & Brassel, G. *Fossilien im Hunsrückschiefer: Dokumente des Meereslebens im Devon*. [Fossils of the Hunsrück Slate: Documents of the sea-life in the Devonian]. Museum Idar-Oberstein (1990).  Bruton & Haas (1999): fig. 18a,b  (Using the collection of Prof. W. Blind)  Bruton, D., & Haas, W. The anatomy and functional morphology of Phacops (Trilobita) from the Hunsrück Slate (Devonian). *Palaeontographica Abteilung A*, 29-75 (1999). | Not mentioned by Stürmer |
| WS 4.1./503 | Hunsrück-  Museum Simmern  2020.057 | This research: Fig. 1k,l [CWS] |  |
| WS 11352c | XRCB | This research: Fig. 2a-d |  |
| WS without number | SCB  XRCB | This research: fig. 1m |  |

Our figures are not taken from these references, except those from Stürmer *et al.* (1980) [1a-c] and Stürmer & Bergström (1973) [Fig. 1n,q] as mentioned in the text. Our figures are own photographs made of the radiographs: SCB = Collection Christoph Bartels, Bochum/Steinmann Institute, University of Bonn.
